# Supplementary material for: Influences on university students’ intention to receive recommended vaccines: a cross-sectional survey
Source: BMJ Open. 2017 Jul 21;7(7):e016544. doi: 10.1136/bmjopen-2017-016544 (PMC5642662; doi:10.1136/bmjopen-2017-016544)
Supplement: Supplementary material 1 [file bmjopen-2017-016544supp001.pdf]

Supplementary File. Questionnaire. Items excluded (q 34-36; 39-41) as they were not designed for the current study.

Influences on students' intention to vaccinate

The NHS recommends a number of vaccinations for individuals of different ages and high-risk groups, free of charge. The NHS childhood vaccination programme offers a range of vaccines for children from 2 months up until they are 18. These vaccines protect children against vaccine-preventable diseases such as measles, human papillomavirus (HPV) and polio. People in the UK are also eligible for some free travel vaccines if they are visiting high-risk areas of the world.

We are interested in understanding students' attitudes towards vaccination.

Please read each of the statements below and show how much you agree or disagree with it by ticking the appropriate box.

* 2. Vaccinations are effective in preventing disease.

Strongly disagree	Disagree	Neither agree nor disagree	Agree	Strongly agree
○	○	○	○	○

* 3. I am concerned about vaccination side effects.

Strongly disagree	Disagree	Neither agree nor disagree	Agree	Strongly agree
○	○	○	○	○

* 4. Vaccinations are helpful.

Strongly disagree	Disagree	Neither agree nor disagree	Agree	Strongly agree
○	○	○	○	○

* 5. I am afraid of vaccinations.

Strongly disagree	Disagree	Neither agree nor disagree	Agree	Strongly agree
○	○	○	○	○

* 6. There are more reasons to have vaccinations than not to have vaccinations.

Strongly disagree	Disagree	Neither agree nor disagree	Agree	Strongly agree
○	○	○	○	○

* 7. I have a responsibility to receive vaccinations for the protection of others.

Strongly disagree	Disagree	Neither agree nor disagree	Agree	Strongly agree
○	○	○	○	○

* 8. People who don't get vaccinated put others at risk.

Strongly disagree	Disagree	Neither agree nor disagree	Agree	Strongly agree
○	○	○	○	○

* 9. More people should be vaccinated against diseases so that outbreaks do not occur.

Strongly disagree	Disagree	Neither agree nor disagree	Agree	Strongly agree
○	○	○	○	○

* 10. Vaccinating is doing the right thing.

Strongly disagree	Disagree	Neither agree nor disagree	Agree	Strongly agree
○	○	○	○	○

* 11. Vaccination is one way to make a positive contribution to your health.

Strongly disagree	Disagree	Neither agree nor disagree	Agree	Strongly agree
○	○	○	○	○

Influences on students' intention to vaccinate

Certain travel vaccinations are available free on the NHS. These vaccines immunize people against certain diseases prevalent in parts of the world (such as typhoid, hepatitis A and cholera). Imagine you are planning to travel to a part of Asia where typhoid is prevalent. Typhoid fever is a bacterial infection that can spread throughout the body affecting many organs. It can be fatal without prompt treatment. A typhoid vaccine is recommended and is available free on the NHS, usually from GP surgeries. You can find out more about typhoid fever and the vaccine here: http://www.nhs.uk/Conditions/Typhoid-fever/Pages/Introduction.aspx.

We will ask you some questions about the typhoid vaccine.

* 12. Have you had the typhoid vaccine in the last 3 years?*(a typhoid booster vaccination is recommended every 3 years if you continue to be at risk of infection).*

- Yes
- No
- Don't know

If *yes*, please imagine that you needed the vaccine for the following questions.

We are interested in what you think the views of others would be.

* 13. My immediate family members would think I should get the vaccine.*For example your Mum, Dad, brothers or sisters.*

Agree (1)	2	3	4	5	6	Disagree (7)
○	○	○	○	○	○	○

* 14. My immediate family members would approve of the vaccine.*For example your Mum, Dad, brothers or sisters.*

Agree (1)	2	3	4	5	6	Disagree (7)
○	○	○	○	○	○	○

* 15. Most of my family members would get the vaccine if it was recommended for them*For example your Mum, Dad, brothers or sisters.*

Likely (1)	2	3	4	5	6	Unlikely (7)
○	○	○	○	○	○	○

* 16. My friends would think I should get the vaccine.

Agree (1)	2	3	4	5	6	Disagree (7)
○	○	○	○	○	○	○

* 17. My friends would approve of the vaccine.

Agree (1)	2	3	4	5	6	Disagree (7)
○	○	○	○	○	○	○

* 18. Most of my friends would get the vaccine if it was recommended for them.

Likely(1)	2	3	4	5	6	Unlikely (7)
○	○	○	○	○	○	○

* 19. My GP would think I should get the vaccine.

Agree (1)	2	3	4	5	6	Disagree (7)
○	○	○	○	○	○	○

* 20. My GP would approve of the vaccine.

Agree (1)	2	3	4	5	6	Disagree (7)
○	○	○	○	○	○	○

* 21. My GP would get the vaccine if it was recommended for him/her.

Likely (1)	2	3	4	5	6	Unlikely (7)
○	○	○	○	○	○	○

Influences on students' intention to vaccinate

We're also interested in how *in control* you would feel about getting the typhoid vaccine.

* 22. Whether or not I got the vaccine would be entirely up to me.

Strongly disagree (1)	2	3	4	5	6	Strongly agree (7)
◯	◯	◯	◯	◯	◯	◯

* 23. How much do you feel that whether you got the vaccine would be beyond your control?

Not at all (1)	2	3	4	5	6	Very much so (7)
◯	◯	◯	◯	◯	◯	◯

Influences on students' intention to vaccinate

* 24. I believe I would have the ability to get the vaccine.

Definitely do not (1)	2	3	4	5	6	Definitely do (7)
◯	◯	◯	◯	◯	◯	◯

* 25. To what extent do you see yourself as being capable of getting the vaccine?

Very unlikely (1)	2	3	4	5	6	Very likely (7)
◯	◯	◯	◯	◯	◯	◯

* 26. How confident are you that you would be able to get the vaccine?

Very unsure (1)	2	3	4	5	6	Very sure (7)
◯	◯	◯	◯	◯	◯	◯

* 27. If it were entirely up to me, I am confident that I would be able to get the vaccine.

Strongly disagree (1)	2	3	4	5	6	Strongly agree (7)
◯	◯	◯	◯	◯	◯	◯

Influences on students' intention to vaccinate

Please tick the box that applies most to you.

* 28. Did you have all the recommended vaccines as a child?

- I had none of the recommended vaccines as a child
- I had a few of the recommended vaccines as a child
- I had most of the recommended vaccines as a child
- I had all of the recommended vaccines as a child
- I don't know what vaccines I had as a child

* 29. Have you ever had a bad reaction to a vaccination?

- Yes
- No
- Don't know

Influences on students' intention to vaccinate

* 30. If *yes*, how serious was your reaction?

- Required medical treatment
- Uncomfortable for more than 1 day (no medical treatment)
- Uncomfortable for less than one day (no medical treatment)
- I don't know

Influences on students' intention to vaccinate

* 31. Are you scared of needles?

Not at all (1)	2	3	4	Very scared (5)
○	○	○	○	○

Influences on students' intention to vaccinate

* 32. If I were recommended a vaccine, I would try to get it.

Strongly disagree (1)	2	3	4	Strongly agree (5)
○	○	○	○	○

* 33. If I were recommended a vaccine, I would intend to get it.

Strongly disagree (1)	2	3	4	Strongly agree (5)
○	○	○	○	○

Influences on students' intention to vaccinate

Now we'd like to ask you some more general questions about your health.

Imagine you had a problem with your health.

37. Would you use any of these sources for information? For sources that you would use, please specify the name of the source. For example, for the internet, you might use the NHS website.

Books

Brochures, pamphlets

Family

Friend/Co-worker

Doctor or health care provider

Internet

Library

Magazines

Newspapers

Telephone information number

Complementary, alternative, or unconventional practitioner

Other

Imagine you have been recommended to have a vaccination as part of the UK Immunisation Schedule.

38. Would you use any of these sources for information on the vaccine? For sources that you would use, please specify the name of the source. For example, for the internet, you might use the NHS website.

Books	
Brochures, pamphlets	
Family	
Friend/Co-worker	
Doctor or health care provider	
Internet	
Library	
Magazines	
Newspapers	
Telephone information number	
Complementary, alternative, or unconventional practitioner	
Other	

Influences on students' intention to vaccinate

We are now going to ask some demographic questions.

* 42. What is your age?

* 43. What is your gender?

- Male
- Female
- Prefer not to say

* 44. Do you have any children?

- Yes
- No
- Prefer not to say

* 45. What is your ethnic group? (Please tick one box)

- White British
- White other *(please specify below)*
- Asian British
- Asian other *(please specify below)*
- Black British
- Black other *(please specify below)*
- Mixed ethnicity British *(please specify below)*
- Mixed ethnicity other *(please specify below)*
- Arab
- Other ethnic group *(please specify below)*
- Prefer not to say

Please specify here, if asked

* 46. Where did you live until the age of 4?

- UK
- Not UK *(please specify below)*
- Both *(please specify below)*

Please specify here, if asked

* 47. Do you have a religion?

- No religion
- Christian (including Church of England, Catholic, Protestant and all other Christian denominations)
- Buddhist
- Hindu
- Jewish
- Muslim
- Sikh
- Prefer not to say
- Any other religion (please specify)

Think of this ladder as representing where people stand in the UK.

At the *top* of the ladder are the people who are best off – those who have the most money, the most education and the most respected jobs. At the *bottom* are the people who are the worst off – who have the least money, least education, and the least respected jobs or no job. The higher up you are on this ladder, the closer you are to the people at the very top; the lower you are, the closer you are to the people at the very bottom.

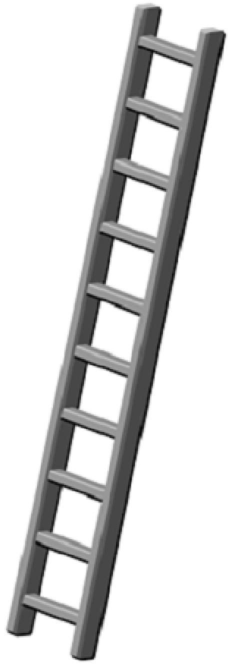

* 48. Now think about your family. Please tell us where you think your family would be on this ladder.

Please tick the box next to the rung that best represents where your family would be on this ladder.

- Top: Rung 10
- Rung 9
- Rung 8
- Rung 7
- Rung 6
- Rung 5
- Rung 4
- Rung 3
- Rung 2
- Bottom: Rung 1

* 49. Where would you place yourself on this ladder?

Please tick the box next to the rung where you think you stand at this time in your life, relative to other people in the UK.

- Top: Rung 10
- Rung 9
- Rung 8
- Rung 7
- Rung 6
- Rung 5
- Rung 4
- Rung 3
- Rung 2
- Bottom: Rung 1
