# Supplementary material for: Influences on university students’ intention to receive recommended vaccines: a cross-sectional survey
Source: BMJ Open. 2017 Jul 21;7(7):e016544. doi: 10.1136/bmjopen-2017-016544 (PMC5642662; doi:10.1136/bmjopen-2017-016544)
Supplement: Author's manuscript [file bmjopen-2017-016544.draft_revisions.pdf]

BMJ Open is committed to open peer review. As part of this commitment we make the peer review history of every article we publish publicly available.

When an article is published we post the peer reviewers' comments and the authors' responses online. We also post the versions of the paper that were used during peer review. These are the versions that the peer review comments apply to.

The versions of the paper that follow are the versions that were submitted during the peer review process. They are not the versions of record or the final published versions. They should not be cited or distributed as the published version of this manuscript.

BMJ Open is an open access journal and the full, final, typeset and author-corrected version of record of the manuscript is available on our site with no access controls, subscription charges or pay-per-view fees (http://bmjopen.bmj.com).

If you have any questions on BMJ Open's open peer review process please email editorial.bmjopen@bmj.com

BMJ Open

Influences on university students' intention to receive recommended vaccines: a cross-sectional survey

Journal:	*BMJ Open*
Manuscript ID	bmjopen-2017-016544
Article Type:	Research
Date Submitted by the Author:	02-Mar-2017

Complete List of Authors:	Landowska, Kate; University College London, Research Department of Behavioural Science and Health<br>Waller, Jo; UCL, Research Department of Behavioural Science and Health<br>Bedford, Helen; University College London, Institute of Child Health<br>Rockliffe, Lauren; UCL, Research Department of Behavioural Science and Health<br>Forster, Alice; UCL, Research Department of Behavioural Science and Health

**Primary Subject Heading**:	Public health
Secondary Subject Heading:	Infectious diseases
Keywords:	Infection control < INFECTIOUS DISEASES, PREVENTIVE MEDICINE, PUBLIC HEALTH

SCHOLARONE™ Manuscripts

Influences on university students' intention to receive recommended vaccines: a cross-sectional survey

Authors: Kate Landowska, Jo Waller, Helen Bedford, Lauren Rockliffe and Alice S Forster

Corresponding Author Contact Details

Name: Dr Alice S Forster

Postal Address: Research Department of Behavioural Science and Health, UCL, Gower Street, London, WC1E 6BT

Email: alice.forster@ucl.ac.uk

Telephone: 0203 108 3293

Details of authors

Name: Miss Kate Landowska

Department: Research Department of Behavioural Science and Health

Institution: UCL

City: London

Country: United Kingdom

Name: Dr Jo Waller

Department: Research Department of Behavioural Science and Health

Institution: UCL

City: London

Country: United Kingdom

Name: Professor Helen Bedford

Department: Great Ormond Street Institute of Child Health

Institution: UCL

City: London

Country: United Kingdom

Name: Miss Lauren Rockliffe

Department: Research Department of Behavioural Science and Health

Institution: UCL

City: London

Country: United Kingdom

Name: Dr Alice Forster

Department: Research Department of Behavioural Science and Health

For peer review only

Institution: UCL

City: London

Country: United Kingdom

Up to five keywords relevant to the content of the manuscript: Immunization, University student, Great Britain, Theory of Planned Behaviour

Word Count (excluding title page, abstract, references, figures and tables): 1,492

Number of Tables: 2

Number of References: 20

For peer review only

ABSTRACT

Objectives: To explore predictors of university students' intention to receive a recommended vaccine, and the main sources of vaccine-related information accessed by university students.

Setting: Participants were recruited from University College London (UK) in summer 2015.

Participants: 177 university students participated. The majority of participants were female (58%), White (68%) and had no religion (58%). Participants were aged 18 to 42 (mean age = 23.6).

Primary and secondary outcome measures: Primary outcome measures included vaccine attitude, perceived subjective norm, perceived behavioural control, perceived self-efficacy, past receipt of recommended childhood vaccines, perceived adverse reaction to past vaccination and needle fear. As a secondary outcome sources of vaccine-related information were assessed.

Results: Students classified as high intenders were more likely to have received all recommended childhood vaccines (OR 3.57; 95% CI 1.21 to 10.59; p=0.022); be less afraid of needles (OR 2.44; 95% CI 1.12 to 5.36; p=0.026); and to have lived in the UK until at least the age of four compared to those not living in the UK until at least the age of four (OR 0.39; 95% CI 0.18 to 0.83; p=0.015) and those who lived both in the UK and elsewhere (OR 0.42; 95% CI 0.04 to 4.06; p=0.424). The multi-variable model explained 25.5% of variance in intention to receive a recommended vaccine. The Internet was the most commonly reported source of vaccination information.

Conclusions: Findings provide an indication of the factors that may need to be addressed by interventions aiming to increase uptake of recommended vaccines in a university population. Future research is recommended using a prospective cohort design.

Trial registration: N/A

STRENGTHS AND LIMITATIONS OF THIS STUDY

- This study was sufficiently powered to identify predictors of intention to vaccinate in a university student population.
- It was not possible to determine the response rate to the survey; respondents may have been those with an interest in the subject.
- Findings are correlational so future research should consider using a prospective design.

For peer review only

INTRODUCTION

Vaccine-preventable diseases (VPDs) are associated with significant mortality and morbidity. Certain groups of individuals are more likely to be exposed to particular VPDs. For example, university students are at higher risk of meningitis and septicaemia due to living in close contact in shared accommodation.[1] It is important to maintain high vaccine coverage to control incidence of VPDs and protect high-risk groups. If sufficient numbers of people remain unprotected, disease outbreaks can occur (e.g.[2]). Understanding why some people do not get vaccinated is complex. Some people appear to reject vaccines without question, while others experience uncertainty, which might result in a delay in obtaining the vaccine or rejection of the vaccine.[3-6] Practical barriers can also prevent immunisation.

Previous research has suggested that vaccine uptake is significantly predicted by an individual's intention to vaccinate.[7] The Theory of Planned Behaviour[8] and the Health Belief Model[9] have both been shown to predict intention to vaccinate.[10] Other predictors include perceived self-efficacy (PSE; a person's belief about their capability of accomplishing a task);[11] previous vaccination experience;[12] and needle fear.[13]

There has been little research investigating predictors of vaccine intention among students attending UK universities. It is important to understand how to promote vaccine uptake in this population as in the UK vaccines are recommended specifically for this group if they are unprotected when entering university (for example Meningococcal ACWY is recommended for unvaccinated university entrants). We carried out a survey to identify 1) predictors of university students' intention to receive a recommended vaccine and 2) their main sources of vaccine-related information.

METHODS

In summer 2015, 177 students were recruited via an advertisement email circulated by departmental administrators at University College London (UCL), based on a sample size calculation (f^2 = 0.1;[14] alpha =.05; power =.8; predictors = 10). Potential participants were given the incentive of winning an iPad. The study was approved by the UCL research ethics committee (6613/001).

Respondents completed a 48-item online questionnaire (see Supplementary File). Items were developed from previous surveys.[15-17] Intention to receive a recommended vaccine was measured using two items, responses to which were summed to give a possible score of 2-

For peer review only

10; ‘I would try to get a recommended vaccine’ and ‘I would intend to get a recommended vaccine’ (five-point scale: strongly disagree [1] to strongly agree [5]). Predictor variables measured included vaccine attitude; perceived subjective norm (PSN, whether the respondent thinks that significant others would approve of them getting a vaccine and their motivation to comply with these wishes), perceived behavioural control (PBC, perceived ease in getting a vaccine); perceived self-efficacy (PSE); past receipt of recommended childhood vaccines; perceived adverse reaction to past vaccination; and needle fear. Participants also provided demographic information. Sources of vaccine-related information were assessed using a single-item, modified from the Health Information National Trends Survey, 2013.[18] Participants were not able to proceed with the survey if they failed to provide a response to an item. This ensured no data were missing.

Data were analysed using SPSS V.19. The score for intention to receive a recommended vaccine was skewed, so a binary outcome was created using a median split, with those scoring 2–9 (out of 10) classified as ‘low intenders’ and those scoring 10 as ‘high intenders’. Predictor variables were recoded into binary variables using a median split including attitude (positive/negative), PSN, PBC and PSE (all high/low) and needle fear (scared/not scared). Response options for past vaccine receipt was grouped into ‘not all childhood vaccines received’ or ‘all childhood vaccines received’.

A logistic regression analysis was performed to examine the association between predictor variables and vaccine intention. All factors showing significant associations with intention on univariable analyses ($p<0.05$) were entered into a multi-variable model. For the secondary question, we tabulated the number of students who reported using each source of vaccine-related information.

RESULTS

Participants

A total of 177 students completed the vaccine intention questions [57.6% female; 37.9% male; 4.5% prefer not to say/unknown; aged 18-42 (mean age = 23.61, SD = 4.43)]. The majority of respondents were White (68%) and self-defined as having no religion (58%). Approximately 50% had lived in the UK until at least the age of four, likely due to a high proportion of international students completing the survey. It was not possible to estimate a response rate due to the method of contacting potential participants.

For peer review only

Predictors of vaccine intention

Factors significantly associated with intention were entered into a multi-variable model, which explained 25.5% of variance in intention to receive a recommended vaccine (Table 1). Past vaccine receipt, needle fear and country of residence until at least the age of 4 remained significant predictors of vaccine intention in the model (p=0.022, p=0.026 and p=0.015, respectively). Participants who had received all recommended childhood vaccines were over three times more likely to be high vaccine intenders than respondents who had not [Odds ratio (OR) 3.57, 95% CI 1.21-10.59]. Students who were less afraid of needles were over two times more likely to be high vaccine intenders than students who were more afraid [OR 2.44, 95% CI 1.12-5.36]. Moreover, participants who did not live in the UK until the age of four were significantly less likely to be high vaccine intenders than participants who had [OR 0.39, 95% CI 0.18-0.83].

For peer review only

Table 1 Behavioural and demographic predictors of having a high intention to receive a recommended vaccine (n=177[a])

Variable	n (%) with high intention to receive a recommended vaccine [b]	Uni-variable analyses: Odds ratio (95% CI)	Uni-variable analyses: *p*	Multi-variable analysis (n=177*): Odds ratio (95% CI)	Multi-variable analysis: *p*
Attitude					
Negative attitude	25 (27.5)	1.00		1.0	
Positive attitude	54 (62.8)	4.46 (2.36-8.41)	≤0.001	1.74 (0.75-4.05)	0.196
Perceived Subjective Norm (PSN)					
Low PSN	28 (30.4)	1.00		1.0	
High PSN	51 (60.0)	3.43 (1.84-6.38)	≤0.001	2.22 (0.92-5.35)	0.077
Perceived behavioural control (PBC)					
Low PBC	40 (40.4)	1.00			
High PBC	39 (50.0)	1.48 (0.81-2.68)	0.203		
Perceived Self-efficacy (PSE)					
Low PSE	29 (32.6)	1.00		1.0	
High PSE	50 (56.8)	2.72 (1.48-5.02)	0.001	1.07 (0.46-2.51)	0.876
Past receipt of recommended childhood vaccines					
Not all recommended childhood vaccines	6 (15.8)	1.00		1.0	
All recommended childhood vaccines	72 (53.3)	6.1 (2.39-15.53)	≤0.001	3.57 (1.21-10.59)	0.022
Past perceived adverse reaction to vaccination					
Bad reaction	4 (22.2)	1.00		1.0	
No bad reaction	72 (47.7)	3.19 (1.00-10.14)	0.049	3.53 (0.92-13.53)	0.066
Needle Fear					
Scared of needles	40 (36.0)	1.00		1.0	
Not scared of needles	39 (59.1)	2.56 (1.37-4.79)	0.003	2.44 (1.12-5.36)	0.026
Age					
18-25	59 (46.8)	1.00			
26-33	15 (39.5)	0.74 (0.35-1.55)	0.426		
34-42	2 (28.6)	0.45 (0.09-2.43)	0.356		
Gender					
Male	27 (40.3)	1.00			
Female	49 (48.0)	1.37 (0.73-2.56)	0.323		
Ethnicity					
White British/Other	59 (48.8)	1.00			
Asian British/Other	10 (34.5)	0.55 (0.24-1.29)	0.169		
Black British/Other	0 (0)	-	-		
Mixed Ethnicity British/Other	4 (50.0)	1.05 (0.25-4.40)	0.946		
Other Ethnic Group	1 (16.7)	0.21 (0.02-1.85)	0.160		
Country of residence until the age of 4					
UK	51 (58.0)	1.00		1.0	
Not UK	23 (29.9)	0.31 (0.16-0.59)	≤0.001	0.39 (0.18-0.83)	0.015
Both	2 (33.3)	0.36 (0.06-2.09)	0.256	0.42 (0.04-4.06)	0.424
Religion					
No religion	47 (46.1)	1.00			
Christian	18 (41.9)	0.84 (0.41-1.73)	0.641		
Other religion	9 (40.9)	0.81 (0.32-2.06)	0.659		
Deprivation (possible range: 2-20)	Mean = 9.78, SD = 2.97	1.11 (0.10-1.23)	0.06		

[a] Note that *n* differs slightly between items due to missing data. [b] Unless specified.

For peer review only

Information sources

The five most commonly reported vaccine information sources were the Internet (66.7%); doctor or healthcare provider (56.5%); family (24.9%); brochures or pamphlets (23.7%); and friend or co-worker (14.7%). All other sources were reported by <4% of students (Table 2). The most commonly specified websites were the National Health Service (NHS), search engines (e.g. Google) and Health/Medical information websites (e.g. webMD; patient.co.uk).

Table 2 Sources of vaccine-related information (*n*=177[a])

Source	n (%) that has used source for vaccine-related information
Internet	118 (66.67)
Doctor or healthcare provider	100 (56.50)
Family	44 (24.86)
Brochures, pamphlets	42 (23.73)
Friend / co-worker	26 (14.69)
Books	6 (3.39)
Newspapers	6 (3.39)
Library	4 (2.26)
Magazines	4 (2.26)
Telephone information number	4 (2.26)
Complementary, alternative, or unconventional practitioner	2 (1.13)
Other (examples reported: university student psychological services; organic and ethical health forums; pharmacy; someone with a similar health problem)	3 (1.69)

[a] Column n >177 as multiple responses were permitted.

For peer review only - http://bmjopen.bmj.com/site/about/guidelines.xhtml

For peer review only

DISCUSSION

This study explored influences on university students' intentions to receive a recommended vaccine. We found that past vaccine receipt, needle fear and country of residence until at least the age of four were significant predictors of vaccine intention.

Past vaccine receipt had the strongest influence on intention. Students who had received all recommended childhood vaccines were over three times more likely to be high vaccine intenders than students who had not. This is consistent with a previous systematic review which found that receiving all childhood vaccinations was a positive predictor for human papillomavirus (HPV) vaccine initiation in teenage girls.[12]

Furthermore, students who were less afraid of needles were more than twice as likely to be high vaccine intenders than students who were more afraid. This supports a previous study which reported needle fear to be a primary reason for vaccine avoidance among adolescent boys.[13] Interventions addressing vaccine uptake in a student population could therefore benefit from focusing on psychological targets such as reducing needle fear by using interventions to reduce pain on immunisation. Moreover, interventions could target students who did not complete their childhood immunisation schedules.

Participants who did not live in the UK before the age of four were significantly less likely to be high vaccine intenders compared with participants who had. To our knowledge there is no previous research investigating the influence of country of childhood residence on intention to vaccinate as an adult. This could be an interesting direction for future research which could assess differences in immunisation schedules between countries and the effects of disparities on uptake.

The five most commonly reported sources of vaccine-related information were the Internet; doctor/healthcare provider; family; brochures/pamphlets; and friend/co-worker. The listed sources are consistent with a critical appraisal reviewing 38 studies, although their order of preference is not.[19] Unlike the appraisal, current findings indicate that the most common vaccine-related source for students is the Internet. This may be because our sample population was younger. Younger adults have been found to use technology more frequently than older adults.[20] However, the types of website accessed are similar in both our sample and samples in the appraisal. Official websites (i.e. NHS website, Web MD) were reported more frequently than blogs and Wikipedia. No students reported social media as a source of online vaccine-related health information. Campaigns could therefore benefit from

For peer review only - http://bmjopen.bmj.com/site/about/guidelines.xhtml

disseminating information on official webpages. Additionally, university students in the UK often live away from home so re-register with a local health provider. Healthcare providers should use this opportunity to provide information in consultations and use pamphlets as a tool to offer further information.

There are several limitations to this study. Due to the cross-sectional design, findings are correlational. It is therefore difficult to infer the direction of causality between outcome and predictor variables. A future prospective cohort design investigating factors predicting vaccine uptake could therefore be insightful. Furthermore, intention to vaccinate in the present study may not be directly predictive of behaviour. Previous research has demonstrated an intention-behaviour gap.[7] Moreover, we cannot be sure of the response rate and there may have been a response bias towards students more interested in this subject. However, a strength of the study is that the sample was large and so we can draw useful conclusions about this sample.

These findings provide an indication of where future interventions to promote vaccines to a university student population might usefully focus.

DATA SHARING STATEMENT

Data is available on request from the corresponding author.

COMPETING INTERESTS

None

FUNDING

AF and LR are funded by a Cancer Research UK – BUPA cancer prevention fellowship awarded to AF (C49896/A17429). JW is funded by a Cancer Research UK Career Development Fellowship (C7492/A17219).

STATEMENT OF CONTRIBUTION

KL, AF, JW and HB developed the study idea and design. KL collected study data and conducted the analysis. AF, JW and HB assisted with data analysis. KL, AF, JW, HB and LR contributed to the write-up and interpretation of study data.

For peer review only - http://bmjopen.bmj.com/site/about/guidelines.xhtml

REFERENCES

1 Read R, Baxter D, Chadwick DR, et al. Effect of a quadrivalent meningococcal ACWY glycoconjugate or a serogroup B meningococcal vaccine on meningococcal carriage: an observer-blind, phase 3 randomised clinical trial. *Lancet* 2014;384:2123-31.

2 Lacobucci G. Wales sets up drop-in vaccination clinics to tackle measles outbreak. *BMJ* 2013;346:f2452.

3 MacDonald N. Vaccine hesitancy: Definition, scope and determinants. *Vaccine* 2015;33:4161-64.

4 Robison S, Groom H, Young C. Frequency of alternative immunization schedule use in a metropolitan area. *Pediatrics* 2012;130:32-38.

5 Samad L, Tate AR, Dezateux C, et al. Differences in risk factors for partial and no immunisation in the first year of life: prospective cohort study. *BMJ* 2006;332:1312-13.

6 Larson H, Jarrett C, Eckersberger E, et al. Understanding vaccine hesitancy around vaccines and vaccination from a global perspective: a systematic review of published literature, 2007–2012. *Vaccine* 2014;32:2150-59.

7 Juraskova I, Bari RA, O'Brien MT, et al. HPV vaccine promotion: does referring to both cervical cancer and genital warts affect intended and actual vaccination behavior? *Womens Health Issues* 2011;21:71-79.

8 Ajzen I. The theory of planned behavior. *Organ Behav Hum Decis Process* 1991;50:179-211.

9 Rosenstock I. Why people use health services. *Milbank Q* 1966;44:94-127.

10 Gerend M, Shepherd JE. Predicting human papillomavirus vaccine uptake in young adult women: Comparing the health belief model and theory of planned behavior. *Ann Behav Med* 2012;44:171-80.

For peer review only - http://bmjopen.bmj.com/site/about/guidelines.xhtml

11 Bandura A. The explanatory and predictive scope of self-efficacy theory. *J Soc Clin Psychol* 1986;4:359-73.

12 Kessels S, Marshall HS, Watson M, et al. Factors associated with HPV vaccine uptake in teenage girls: a systematic review. *Vaccine* 2012;30:3546-56.

13 Forster AS, Marlow LA, Wardle J, et al. Interest in having HPV vaccination among adolescent boys in England. *Vaccine* 2012;30:4505-10.

14 Bowyer H, Forster AS, Marlow LA, et al. Predicting human papillomavirus vaccination behaviour among adolescent girls in England: results from a prospective survey. *J Fam Plann Reprod Health Care* 2014;40:14-22.

15 Fishbein M, Ajzen I. Predicting and changing behavior: The reasoned action approach. New York: Taylor & Francis 2011.

16 Marlow LA, Waller J, Wardle J. Parental attitudes to pre-pubertal HPV vaccination. *Vaccine* 2007;25:1945-52.

17 Armitage CJ, Conner M. Distinguishing Perceptions of Control From Self-Efficacy: Predicting Consumption of a Low-Fat Diet Using the Theory of Planned Behavior. *J Appl Soc Psychol* 1999;29:72-90.

18 National Institutes of Health USDoHaHS. Health Information National Trends Survey. 2013. http://hints.cancer.gov/docs/Instruments/HINTS_4_Cycle_3_English_Annotated_508c_3_21_2014.pdf (accessed 4th July 2016).

19 Yaqub O, Castle-Clarke S, Sevdalis N, et al. Attitudes to vaccination: A critical review. *Soc Sci Med* 2014;112:1-11.

20 Eisma R, Dickinson A, Goodman J, et al. Early user involvement in the development of information technology-related products for older people. *Univers Access Inform Soc* 2004;3:131-40.

For peer review only - http://bmjopen.bmj.com/site/about/guidelines.xhtml

Supplementary File. Questionnaire. Items excluded (q 34-36; 39-41) as they were not designed for the current study.

Influences on students' intention to vaccinate

The NHS recommends a number of vaccinations for individuals of different ages and high-risk groups, free of charge. The NHS childhood vaccination programme offers a range of vaccines for children from 2 months up until they are 18. These vaccines protect children against vaccine-preventable diseases such as measles, human papillomavirus (HPV) and polio. People in the UK are also eligible for some free travel vaccines if they are visiting high-risk areas of the world.

We are interested in understanding students' attitudes towards vaccination.

Please read each of the statements below and show how much you agree or disagree with it by ticking the appropriate box.

* 2. Vaccinations are effective in preventing disease.

Strongly disagree	Disagree	Neither agree nor disagree	Agree	Strongly agree
○	○	○	○	○

* 3. I am concerned about vaccination side effects.

Strongly disagree	Disagree	Neither agree nor disagree	Agree	Strongly agree
○	○	○	○	○

* 4. Vaccinations are helpful.

Strongly disagree	Disagree	Neither agree nor disagree	Agree	Strongly agree
○	○	○	○	○

* 5. I am afraid of vaccinations.

Strongly disagree	Disagree	Neither agree nor disagree	Agree	Strongly agree
○	○	○	○	○

* 6. There are more reasons to have vaccinations than not to have vaccinations.

Strongly disagree	Disagree	Neither agree nor disagree	Agree	Strongly agree
○	○	○	○	○

* 7. I have a responsibility to receive vaccinations for the protection of others.

Strongly disagree	Disagree	Neither agree nor disagree	Agree	Strongly agree
○	○	○	○	○

* 8. People who don't get vaccinated put others at risk.

Strongly disagree	Disagree	Neither agree nor disagree	Agree	Strongly agree
○	○	○	○	○

* 9. More people should be vaccinated against diseases so that outbreaks do not occur.

Strongly disagree	Disagree	Neither agree nor disagree	Agree	Strongly agree
○	○	○	○	○

* 10. Vaccinating is doing the right thing.

Strongly disagree	Disagree	Neither agree nor disagree	Agree	Strongly agree
○	○	○	○	○

* 11. Vaccination is one way to make a positive contribution to your health.

Strongly disagree	Disagree	Neither agree nor disagree	Agree	Strongly agree
○	○	○	○	○

Influences on students' intention to vaccinate

Certain travel vaccinations are available free on the NHS. These vaccines immunize people against certain diseases prevalent in parts of the world (such as typhoid, hepatitis A and cholera). Imagine you are planning to travel to a part of Asia where typhoid is prevalent. Typhoid fever is a bacterial infection that can spread throughout the body affecting many organs. It can be fatal without prompt treatment. A typhoid vaccine is recommended and is available free on the NHS, usually from GP surgeries. You can find out more about typhoid fever and the vaccine here: http://www.nhs.uk/Conditions/Typhoid-fever/Pages/Introduction.aspx.

We will ask you some questions about the typhoid vaccine.

* 12. Have you had the typhoid vaccine in the last 3 years?*(a typhoid booster vaccination is recommended every 3 years if you continue to be at risk of infection).*

- Yes
- No
- Don't know

If *yes*, please imagine that you needed the vaccine for the following questions.

We are interested in what you think the views of others would be.

* 13. My immediate family members would think I should get the vaccine.*For example your Mum, Dad, brothers or sisters.*

Agree (1)	2	3	4	5	6	Disagree (7)
○	○	○	○	○	○	○

* 14. My immediate family members would approve of the vaccine.*For example your Mum, Dad, brothers or sisters.*

Agree (1)	2	3	4	5	6	Disagree (7)
○	○	○	○	○	○	○

* 15. Most of my family members would get the vaccine if it was recommended for them*For example your Mum, Dad, brothers or sisters.*

Likely (1)	2	3	4	5	6	Unlikely (7)
○	○	○	○	○	○	○

* 16. My friends would think I should get the vaccine.

Agree (1)	2	3	4	5	6	Disagree (7)
○	○	○	○	○	○	○

* 17. My friends would approve of the vaccine.

Agree (1)	2	3	4	5	6	Disagree (7)

* 18. Most of my friends would get the vaccine if it was recommended for them.

Likely(1)	2	3	4	5	6	Unlikely (7)

* 19. My GP would think I should get the vaccine.

Agree (1)	2	3	4	5	6	Disagree (7)

* 20. My GP would approve of the vaccine.

Agree (1)	2	3	4	5	6	Disagree (7)

* 21. My GP would get the vaccine if it was recommended for him/her.

Likely (1)	2	3	4	5	6	Unlikely (7)

Influences on students' intention to vaccinate

We're also interested in how *in control* you would feel about getting the typhoid vaccine.

* 22. Whether or not I got the vaccine would be entirely up to me.

Strongly disagree (1)	2	3	4	5	6	Strongly agree (7)
○	○	○	○	○	○	○

* 23. How much do you feel that whether you got the vaccine would be beyond your control?

Not at all (1)	2	3	4	5	6	Very much so (7)
○	○	○	○	○	○	○

Influences on students' intention to vaccinate

* 24. I believe I would have the ability to get the vaccine.

Definitely do not (1)	2	3	4	5	6	Definitely do (7)
○	○	○	○	○	○	○

* 25. To what extent do you see yourself as being capable of getting the vaccine?

Very unlikely (1)	2	3	4	5	6	Very likely (7)
○	○	○	○	○	○	○

* 26. How confident are you that you would be able to get the vaccine?

Very unsure (1)	2	3	4	5	6	Very sure (7)
○	○	○	○	○	○	○

* 27. If it were entirely up to me, I am confident that I would be able to get the vaccine.

Strongly disagree (1)	2	3	4	5	6	Strongly agree (7)
○	○	○	○	○	○	○

Influences on students' intention to vaccinate

Please tick the box that applies most to you.

* 28. Did you have all the recommended vaccines as a child?

- I had none of the recommended vaccines as a child
- I had a few of the recommended vaccines as a child
- I had most of the recommended vaccines as a child
- I had all of the recommended vaccines as a child
- I don't know what vaccines I had as a child

* 29. Have you ever had a bad reaction to a vaccination?

- Yes
- No
- Don't know

Influences on students' intention to vaccinate

* 30. If *yes*, how serious was your reaction?

- Required medical treatment
- Uncomfortable for more than 1 day (no medical treatment)
- Uncomfortable for less than one day (no medical treatment)
- I don't know

Influences on students' intention to vaccinate

* 31. Are you scared of needles?

Not at all (1)	2	3	4	Very scared (5)
○	○	○	○	○

Influences on students' intention to vaccinate

* 32. If I were recommended a vaccine, I would try to get it.

Strongly disagree (1)	2	3	4	Strongly agree (5)
○	○	○	○	○

* 33. If I were recommended a vaccine, I would intend to get it.

Strongly disagree (1)	2	3	4	Strongly agree (5)
○	○	○	○	○

Influences on students' intention to vaccinate

Now we'd like to ask you some more general questions about your health.

Imagine you had a problem with your health.

37. Would you use any of these sources for information? For sources that you would use, please specify the name of the source. For example, for the internet, you might use the NHS website.

Books	
Brochures, pamphlets	
Family	
Friend/Co-worker	
Doctor or health care provider	
Internet	
Library	
Magazines	
Newspapers	
Telephone information number	
Complementary, alternative, or unconventional practitioner	
Other	

Imagine you have been recommended to have a vaccination as part of the UK Immunisation Schedule.

38. Would you use any of these sources for information on the vaccine? For sources that you would use, please specify the name of the source. For example, for the internet, you might use the NHS website.

Books	
Brochures, pamphlets	
Family	
Friend/Co-worker	
Doctor or health care provider	
Internet	
Library	
Magazines	
Newspapers	
Telephone information number	
Complementary, alternative, or unconventional practitioner	
Other	

Influences on students' intention to vaccinate

We are now going to ask some demographic questions.

* 42. What is your age?

* 43. What is your gender?

- Male
- Female
- Prefer not to say

* 44. Do you have any children?

- Yes
- No
- Prefer not to say

* 45. What is your ethnic group? (Please tick one box)

- White British
- White other *(please specify below)*
- Asian British
- Asian other *(please specify below)*
- Black British
- Black other *(please specify below)*
- Mixed ethnicity British *(please specify below)*
- Mixed ethnicity other *(please specify below)*
- Arab
- Other ethnic group *(please specify below)*
- Prefer not to say

Please specify here, if asked

* 46. Where did you live until the age of 4?

- UK
- Not UK *(please specify below)*
- Both *(please specify below)*

Please specify here, if asked

* 47. Do you have a religion?

- No religion
- Christian (including Church of England, Catholic, Protestant and all other Christian denominations)
- Buddhist
- Hindu
- Jewish
- Muslim
- Sikh
- Prefer not to say
- Any other religion (please specify)

Think of this ladder as representing where people stand in the UK.

At the *top* of the ladder are the people who are best off – those who have the most money, the most education and the most respected jobs. At the *bottom* are the people who are the worst off – who have the least money, least education, and the least respected jobs or no job. The higher up you are on this ladder, the closer you are to the people at the very top; the lower you are, the closer you are to the people at the very bottom.

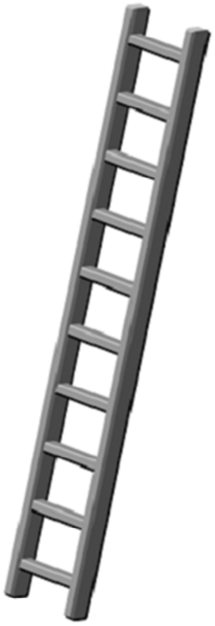

* 48. Now think about your family. Please tell us where you think your family would be on this ladder.

Please tick the box next to the rung that best represents where your family would be on this ladder.

- Top: Rung 10
- Rung 9
- Rung 8
- Rung 7
- Rung 6
- Rung 5
- Rung 4
- Rung 3
- Rung 2
- Bottom: Rung 1

* 49. Where would you place yourself on this ladder?

Please tick the box next to the rung where you think you stand at this time in your life, relative to other people in the UK.

- Top: Rung 10
- Rung 9
- Rung 8
- Rung 7
- Rung 6
- Rung 5
- Rung 4
- Rung 3
- Rung 2
- Bottom: Rung 1

BMJ Open

Influences on university students' intention to receive recommended vaccines: a cross-sectional survey

Journal:	*BMJ Open*
Manuscript ID	bmjopen-2017-016544.R1
Article Type:	Research
Date Submitted by the Author:	31-May-2017

Complete List of Authors:	Landowska, Kate; University College London, Research Department of Behavioural Science and Health<br>Waller, Jo; UCL, Research Department of Behavioural Science and Health<br>Bedford, Helen; University College London, Institute of Child Health<br>Rockliffe, Lauren; UCL, Research Department of Behavioural Science and Health<br>Forster, Alice; UCL, Research Department of Behavioural Science and Health

**Primary Subject Heading**:	Public health
Secondary Subject Heading:	Infectious diseases
Keywords:	Infection control < INFECTIOUS DISEASES, PREVENTIVE MEDICINE, PUBLIC HEALTH

SCHOLARONE™ Manuscripts

For peer review only

Influences on university students' intention to receive recommended vaccines: a cross-sectional survey

Authors: Kate Landowska, Jo Waller, Helen Bedford, Lauren Rockliffe and Alice S Forster

Corresponding Author Contact Details

Name: Dr Alice S Forster

Postal Address: Research Department of Behavioural Science and Health, UCL, Gower Street, London, WC1E 6BT

Email: alice.forster@ucl.ac.uk

Telephone: 0203 108 3293

Details of authors

Name: Miss Kate Landowska

Department: Research Department of Behavioural Science and Health

Institution: UCL

City: London

Country: United Kingdom

Name: Dr Jo Waller

Department: Research Department of Behavioural Science and Health

Institution: UCL

City: London

Country: United Kingdom

Name: Professor Helen Bedford

Department: Great Ormond Street Institute of Child Health

Institution: UCL

City: London

Country: United Kingdom

Name: Miss Lauren Rockliffe

Department: Research Department of Behavioural Science and Health

Institution: UCL

City: London

Country: United Kingdom

Name: Dr Alice Forster

Department: Research Department of Behavioural Science and Health

For peer review only

Institution: UCL

City: London

Country: United Kingdom

Up to five keywords relevant to the content of the manuscript: Immunization, University student, Great Britain, Theory of Planned Behaviour

Word Count (excluding title page, abstract, references, figures and tables): 2,035

Number of Tables: 2

Number of References: 25

For peer review only

ABSTRACT

Objectives: To explore predictors of university students' intention to receive a recommended vaccine, and the main sources of vaccine-related information accessed by university students.

Setting: Participants were recruited from University College London (UK) in summer 2015.

Participants: 177 university students participated. The majority of participants were female (58%), White (68%) and had no religion (58%). Participants were aged 18 to 42 (mean age = 23.6).

Primary and secondary outcome measures: Primary outcome measures included vaccine attitude, perceived subjective norm, perceived behavioural control, perceived self-efficacy, past receipt of recommended childhood vaccines, perceived adverse reaction to past vaccination and needle fear. As a secondary outcome sources of vaccine-related information were assessed.

Results: Students classified as high intenders were more likely to have received all recommended childhood vaccines (OR 3.57; 95% CI 1.21 to 10.59; p=0.022); be less afraid of needles (OR 2.44; 95% CI 1.12 to 5.36; p=0.026); and to have lived in the UK until at least the age of four compared to those not living in the UK until at least the age of four (OR 0.39; 95% CI 0.18 to 0.83; p=0.015) and those who lived both in the UK and elsewhere (OR 0.42; 95% CI 0.04 to 4.06; p=0.424). The multi-variable model explained 25.5% of variance in intention to receive a recommended vaccine. The internet was the most commonly reported source of vaccination information.

Conclusions: Findings provide an indication of the factors that may need to be addressed by interventions aiming to increase uptake of recommended vaccines in a university population. Future research is recommended using a prospective cohort design.

Trial registration: N/A

STRENGTHS AND LIMITATIONS OF THIS STUDY

- This study was sufficiently powered to identify predictors of intention to vaccinate in a university student population.
- It was not possible to determine the response rate to the survey; participants may have been those with an interest in the subject.
- Findings are correlational so future research should consider using a prospective design.

For peer review only

INTRODUCTION

Vaccine-preventable diseases (VPDs) are associated with significant mortality and morbidity. Certain groups of individuals are more likely to be exposed to particular VPDs. For example, university students are at higher risk of meningitis and septicaemia due to living in close contact in shared accommodation.[1] It is important to maintain high vaccine coverage to control incidence of VPDs and protect high-risk groups. If sufficient numbers of people remain unprotected, disease outbreaks can occur (e.g.[2]). Understanding why some people do not get vaccinated is complex. Some people appear to reject vaccines without question, while others experience uncertainty, which might result in a delay in obtaining the vaccine or rejection of the vaccine.[3-6] Practical barriers can also prevent immunisation.

Previous research has suggested that vaccine uptake is significantly predicted by an individual's intention to vaccinate.[7] The Theory of Planned Behaviour[8] and the Health Belief Model[9] have both been shown to predict intention to vaccinate.[10] Other predictors include perceived self-efficacy (PSE; a person's belief about their capability of accomplishing a task);[11] previous vaccination experience;[12] and needle fear.[13]

There has been little research investigating predictors of vaccine intention among students attending UK universities. It is important to understand how to promote vaccine uptake in this population as in the UK vaccines are recommended specifically for this group if they are unprotected when entering university. The main vaccination recommended for university entrants since 2015 is the Meningococcal ACWY vaccination, which protects against life threatening meningitis and septicaemia and was introduced in response to the rapid increase in MenW cases. Rates of nasopharyngeal carriage of meningococcal organisms are particularly high in university students and the close contact they have with one another due to their living arrangements and lifestyle puts them at increased risk of disease.[14] It is also important that students have had two doses of MMR vaccine. Recent outbreaks of measles in England have mainly affected unimmunised teenagers and young adults who may have missed out on the vaccine at the height of public concerns over its safety in the early 2000s.[15, 16]

In this study we carried out a survey to identify 1) predictors of university students' intention to receive a recommended vaccine and 2) their main sources of vaccine-related information.

For peer review only - http://bmjopen.bmj.com/site/about/guidelines.xhtml

METHODS

This study was conducted between June and July 2015. 177 students were recruited via an advertisement email circulated by administrators in 39 departments at University College London (UCL), based on a sample size calculation (f^2 = 0.1;[17] alpha =.05; power =.8; predictors = 10). This calculation suggested that a minimum sample size of 91 participants would be required for the study to be sufficiently powered. Potential participants were given the incentive of being entered into a prize draw to win an iPad. The study was approved by the UCL research ethics committee (6613/001).

Participants completed a 48-item online survey (see Supplementary File). Items were developed from previous surveys.[18-20] These surveys were selected as they investigated predictors (i.e. Theory of Planned Behaviour constructs) of vaccine acceptance and positive behaviour change. Intention to receive a recommended vaccine was measured using two items, responses to which were summed to give a possible score of 2-10; ‘I would try to get a recommended vaccine’ and ‘I would intend to get a recommended vaccine’ (five-point scale: strongly disagree [1] to strongly agree [5]). Predictor variables measured included vaccine attitude; perceived subjective norm (PSN, whether the respondent thinks that significant others would approve of them getting a vaccine and their motivation to comply with these wishes), perceived behavioural control (PBC, perceived ease in getting a vaccine); perceived self-efficacy (PSE); past receipt of recommended childhood vaccines; perceived adverse reaction to past vaccination; and needle fear. Participants also provided demographic information. Sources of vaccine-related information were assessed using a single-item, modified from the Health Information National Trends Survey, 2013.[21] Participants were not able to proceed with the survey if they failed to provide a response to an item. This ensured no data were missing.

Informed consent was gained from all participants at the start of the survey by means of an online consent form. Participants could only proceed to complete the survey once the consent form had been completed.

Data were analysed using SPSS V.19. The score for intention to receive a recommended vaccine was skewed, so a binary outcome was created using a median split, with those scoring 2–9 (out of 10) classified as ‘low intenders’ and those scoring 10 as ‘high intenders’. Predictor variables were recoded into binary variables using a median split including attitude (positive/negative), PSN, PBC and PSE (all high/low) and needle fear (scared/not scared). Response options for past vaccine receipt was grouped into ‘not all childhood vaccines received’ or ‘all childhood vaccines received’.

For peer review only - http://bmjopen.bmj.com/site/about/guidelines.xhtml

A logistic regression analysis was performed to examine the association between predictor variables and vaccine intention. All factors showing significant associations with intention on univariable analyses (p<0.05) were entered into a multi-variable model. For the secondary question, we tabulated the number of students who reported using each source of vaccine-related information. As a sensitivity analysis we re-ran the original analysis splitting intention at the median, minus the median absolute deviation (2-8 vs. 9-10).

RESULTS

Participants

A total of 177 students completed the vaccine intention questions [57.6% female; 37.9% male; 4.5% prefer not to say/unknown; aged 18-42 (mean age = 23.61, SD = 4.43)]. The majority of participants were White (68%), followed by Asian (16%), Black (3%), mixed ethnicity (4%) and other (3%). Most participants self-defined as having no religion (58%), followed by Christian (24%) and other religions (12%). Approximately 44% had lived outside the UK until at least the age of four, the period in which childhood vaccinations are offered. This is somewhat higher than the proportion of international students attending UCL, which is 35%. It was not possible to estimate a response rate due to the method of contacting potential participants.

Predictors of vaccine intention

Factors significantly associated with intention were entered into a multi-variable model, which explained 25.5% of variance in intention to receive a recommended vaccine (Table 1). Past vaccine receipt, needle fear and country of residence until at least the age of 4 remained significant predictors of vaccine intention in the model (*p*=0.022, *p*=0.026 and *p*=0.015, respectively). Participants who had received all recommended childhood vaccines were over three times more likely to be high vaccine intenders than participants who had not [Odds ratio (OR) 3.57, 95% CI 1.21-10.59]. Students who were less afraid of needles were over two times more likely to be high vaccine intenders than students who were more afraid [OR 2.44, 95% CI 1.12-5.36]. Moreover, participants who did not live in the UK until the age of four were significantly less likely to be high vaccine intenders than participants who had [OR 0.39, 95% CI 0.18-0.83].

When we re-ran the analyses dichotomising intention at 8 rather than 9, the results were very similar with no change in significance (analyses not shown).

For peer review only - http://bmjopen.bmj.com/site/about/guidelines.xhtml

For peer review only

For peer review only - http://bmjopen.bmj.com/site/about/guidelines.xhtml

Table 1 Behavioural and demographic predictors of having a high intention to receive a recommended vaccine (*n*=177[a])

Variable	n (%) with high intention to receive a recommended vaccine [b]	Uni-variable analyses: Odds ratio (95% CI)	*p*	Multi-variable analysis (*n*=177*): Odds ratio (95% CI)	*p*
Attitude					
Negative attitude	25 (27.5)	1.00		1.0	
Positive attitude	54 (62.8)	4.46 (2.36-8.41)	≤0.001	1.74 (0.75-4.05)	0.196
Perceived Subjective Norm (PSN)					
Low PSN	28 (30.4)	1.00		1.0	
High PSN	51 (60.0)	3.43 (1.84-6.38)	≤0.001	2.22 (0.92-5.35)	0.077
Perceived behavioural control (PBC)					
Low PBC	40 (40.4)	1.00			
High PBC	39 (50.0)	1.48 (0.81-2.68)	0.203		
Perceived Self-efficacy (PSE)					
Low PSE	29 (32.6)	1.00		1.0	
High PSE	50 (56.8)	2.72 (1.48-5.02)	0.001	1.07 (0.46-2.51)	0.876
Past receipt of recommended childhood vaccines					
Not all recommended childhood vaccines	6 (15.8)	1.00		1.0	
All recommended childhood vaccines	72 (53.3)	6.1 (2.39-15.53)	≤0.001	3.57 (1.21-10.59)	0.022
Perceived adverse reaction to past vaccination					
Bad reaction	4 (22.2)	1.00		1.0	
No bad reaction	72 (47.7)	3.19 (1.00-10.14)	0.049	3.53 (0.92-13.53)	0.066
Needle Fear					
Scared of needles	40 (36.0)	1.00		1.0	
Not scared of needles	39 (59.1)	2.56 (1.37-4.79)	0.003	2.44 (1.12-5.36)	0.026
Age					
18-25	59 (46.8)	1.00			
26-33	15 (39.5)	0.74 (0.35-1.55)	0.426		
34-42	2 (28.6)	0.45 (0.09-2.43)	0.356		
Gender					
Male	27 (40.3)	1.00			
Female	49 (48.0)	1.37 (0.73-2.56)	0.323		
Ethnicity					
White British/Other	59 (48.8)	1.00			
Asian British/Other	10 (34.5)	0.55 (0.24-1.29)	0.169		
Black British/Other	0 (0)	-	-		
Mixed Ethnicity British/Other	4 (50.0)	1.05 (0.25-4.40)	0.946		
Other Ethnic Group	1 (16.7)	0.21 (0.02-1.85)	0.160		
Country of residence until the age of 4					
UK	51 (58.0)	1.00		1.0	
Not UK	23 (29.9)	0.31 (0.16-0.59)	≤0.001	0.39 (0.18-0.83)	0.015
Both	2 (33.3)	0.36 (0.06-2.09)	0.256	0.38 (0.04-4.06)	0.424
Religion					
No religion	47 (46.1)	1.00			
Christian	18 (41.9)	0.84 (0.41-1.73)	0.641		
Other religion	9 (40.9)	0.81 (0.32-2.06)	0.659		
Deprivation (possible range: 2-20)	Mean = 9.78, SD = 2.97	1.11 (1.00-1.23)	0.060		

For peer review only

[a] Note that *n* differs slightly between items due to missing data. [b] Unless specified.

For peer review only - http://bmjopen.bmj.com/site/about/guidelines.xhtml

Information sources

The five most commonly reported vaccine information sources were the internet (66.7%); doctor or healthcare provider (56.5%); family (24.9%); brochures or pamphlets (23.7%); and friend or co-worker (14.7%). All other sources were reported by <4% of students (Table 2). The most commonly specified websites were the National Health Service (NHS), search engines (e.g. Google) and Health/Medical information websites (e.g. webMD; patient.co.uk).

Table 2 Sources of vaccine-related information (*n*=177[a])

Source	n (%) that has used source for vaccine-related information
Internet	118 (66.67)
Doctor or healthcare provider	100 (56.50)
Family	44 (24.86)
Brochures, pamphlets	42 (23.73)
Friend / co-worker	26 (14.69)
Books	6 (3.39)
Newspapers	6 (3.39)
Library	4 (2.26)
Magazines	4 (2.26)
Telephone information number	4 (2.26)
Complementary, alternative, or unconventional practitioner	2 (1.13)
Other (examples reported: university student psychological services; organic and ethical health forums; pharmacy; someone with a similar health problem)	3 (1.69)

[a] Column n >177 as multiple responses were permitted.

For peer review only - http://bmjopen.bmj.com/site/about/guidelines.xhtml

DISCUSSION

This study explored influences on university students' intentions to receive a recommended vaccine. We found that past vaccine receipt, needle fear and country of residence until at least the age of four were significant predictors of vaccine intention.

Past vaccine receipt had the strongest influence on intention. Students who had received all recommended childhood vaccines were over three times more likely to be high vaccine intenders than students who had not. This is consistent with a previous systematic review which found that receiving all childhood vaccinations was a positive predictor for human papillomavirus (HPV) vaccine initiation in teenage girls.[12]

Furthermore, students who were less afraid of needles were more than twice as likely to be high vaccine intenders than students who were more afraid. This supports a previous study which reported needle fear to be a primary reason for vaccine avoidance among adolescent boys.[13] Interventions addressing vaccine uptake in a student population could therefore benefit from focusing on psychological targets such as reducing needle fear by using interventions to reduce pain on immunisation. Moreover, interventions could target students who did not complete their childhood immunisation schedules.

Participants who did not live in the UK before the age of four were significantly less likely to be high vaccine intenders compared with participants who had. To our knowledge there is no previous research investigating the influence of country of childhood residence on intention to vaccinate as an adult. This could be an interesting direction for future research which could assess differences in immunisation schedules between countries and the effects of disparities on uptake.

The five most commonly reported sources of vaccine-related information were the internet; doctor/healthcare provider; family; brochures/pamphlets; and friend/co-worker. The popularity of these sources is consistent with a critical appraisal reviewing 38 studies. [22] The findings of this study indicate that the most common vaccine-related source for students is the internet, which corroborates previous research which found the internet to be the most common health and medicine source used in the UK.[23] However, previous studies suggest that the internet is perceived as less trustworthy than sources such as a GP and a medically qualified friend or relative.[23] It is therefore unlikely to supplant the role of these sources, but may be used to supplement them.[24] Official websites (i.e. NHS website, Web MD) were reported more frequently than blogs and Wikipedia. This substantiates previous

For peer review only - http://bmjopen.bmj.com/site/about/guidelines.xhtml

findings suggesting that official websites are trusted more than less well-moderated websites.[22] No students reported social media as a source of online vaccine-related health information. Campaigns could therefore benefit from disseminating information on official webpages. Additionally, university students in the UK often live away from home so re-register with a local health provider. Healthcare providers should use this opportunity to provide information in consultations and use pamphlets as a tool to offer further information.

There are several limitations to this study. Due to the cross-sectional design, findings are correlational. It is therefore difficult to infer the direction of causality between outcome and predictor variables. A future prospective cohort design investigating factors predicting vaccine uptake could therefore be insightful. Intention to vaccinate in the present study may not be directly predictive of behaviour. Previous research has demonstrated an intention-behaviour gap.[7] Moreover, we cannot be sure of the response rate as it is not clear how many university departments circulated the recruitment email. Consequently we are unable to assess non-response bias and there may have been a response bias towards students more interested in this subject. Some survey items relied on participants' knowledge of vaccinations received during childhood and perception of past adverse reactions, which may also have occurred during childhood. There is therefore the potential for inaccuracies in participant reporting, though it could be argued that perceptions of vaccine-related events in childhood would be stronger predictors of subsequent vaccine attitudes than objectively measured events. In addition, the high proportion of participants who lived outside the UK until the age of 4, suggests an over-representation of non-UK born participants. It is therefore uncertain whether the findings would be directly generalizable to the wider student population in the UK. Finally, due to time restrictions we were unable to pilot the survey prior to data collection and there is the potential that some items may lack clarity. A strength of the study is that the sample was large and so we can draw useful conclusions about this sample.

These findings are timely in view of the recent introduction of MenACWY vaccine for 14-18 year olds and for young people up to the age of 25 years attending university for the first time. It is of concern that uptake of the MenACWY vaccine in the first two cohorts of 18 year olds offered the vaccine has not exceeded 39%, leaving many young people at risk of these potentially severe infections.[25] The findings provide an indication of where to focus future interventions to raise university students' awareness of the importance of this and other vaccination programmes.

For peer review only

For peer review only - http://bmjopen.bmj.com/site/about/guidelines.xhtml

DATA SHARING STATEMENT

Data is available on request from the corresponding author.

COMPETING INTERESTS

None

FUNDING

AF and LR are funded by a Cancer Research UK – BUPA cancer prevention fellowship awarded to AF (C49896/A17429). JW is funded by a Cancer Research UK Career Development Fellowship (C7492/A17219).

STATEMENT OF CONTRIBUTION

KL, AF, JW and HB developed the study idea and design. KL collected study data and conducted the analysis. AF, JW, HB and LR assisted with data analysis. KL, AF, JW, HB and LR contributed to the write-up and interpretation of study data.

REFERENCES

1 Read R, Baxter D, Chadwick DR, et al. Effect of a quadrivalent meningococcal ACWY glycoconjugate or a serogroup B meningococcal vaccine on meningococcal carriage: an observer-blind, phase 3 randomised clinical trial. *Lancet* 2014;384:2123-31.

2 Lacobucci G: Wales sets up drop-in vaccination clinics to tackle measles outbreak. *BMJ* 2013;346:f2452.

3 MacDonald N. Vaccine hesitancy: Definition, scope and determinants. *Vaccine* 2015;33:4161-64.

4 Robison S, Groom H, Young C: Frequency of alternative immunization schedule use in a metropolitan area. *Pediatrics* 2012;130:32-38.

5 Samad L, Tate AR, Dezateux C, et al. Differences in risk factors for partial and no immunisation in the first year of life: prospective cohort study. *BMJ* 2006;332:1312-13.

6 Larson H, Jarrett C, Eckersberger E, et al. Understanding vaccine hesitancy around vaccines and vaccination from a global perspective: a systematic review of published literature, 2007–2012. *Vaccine* 2014;32:2150-59.

For peer review only - http://bmjopen.bmj.com/site/about/guidelines.xhtml

7 Juraskova I, Bari RA, O'Brien MT, et al. HPV vaccine promotion: does referring to both cervical cancer and genital warts affect intended and actual vaccination behavior? *Women's Health Issues* 2011;21:71-79.

8 Ajzen I. The theory of planned behavior. *Organ Behav Hum Decis Process* 1991;50:179-211.

9 Rosenstock I. Why people use health services. *Milbank Q* 1966;44:94-127.

10 Gerend M, Shepherd JE. Predicting human papillomavirus vaccine uptake in young adult women: Comparing the health belief model and theory of planned behavior. *Ann Behav Med* 2012;44:171-180.

11 Bandura A. The explanatory and predictive scope of self-efficacy theory. *J Soc Clin Psychol* 1986; 4:359-373.

12 Kessels S, Marshall HS, Watson M, et al. Factors associated with HPV vaccine uptake in teenage girls: a systematic review. *Vaccine* 2012;30:3546-56.

13 Forster AS, Marlow LA, Wardle J, et al. Interest in having HPV vaccination among adolescent boys in England. *Vaccine* 2012;30:4505-10.

14 Public Health England. Guidance on the prevention and management of meningococcal meningitis and septicaemia in higher education institutions. 2016. https://www.gov.uk/government/uploads/system/uploads/attachment_data/file/582511/MenACWY_HEI_Guidelines.pdf (accessed April 2017).

15 Public Health England. Laboratory confirmed cases of measles, mumps and rubella, England: July to September 2016. *Health Protection Report* 2016;10:1-4.

16 Ramsay ME. Measles: the legacy of low vaccine coverage. *Arch Dis Child* 2013;98:752-54.

17 Bowyer H, Forster AS, Marlow LA, et al. Predicting human papillomavirus vaccination behaviour among adolescent girls in England: results from a prospective survey. *J Fam Plann Reprod Health Care* 2014;40:14-22.

18 Fishbein M, Ajzen I. Predicting and changing behavior: The reasoned action approach. New York: Taylor & Francis 2011.

19 Marlow LA, Waller J, Wardle J. Parental attitudes to pre-pubertal HPV vaccination. *Vaccine* 2007;25:1945-52.

20 Armitage CJ, Conner M. Distinguishing Perceptions of Control From Self-Efficacy: Predicting Consumption of a Low-Fat Diet Using the Theory of Planned Behavior. *J Appl Soc Psychol* 1999;29:72-90.

21 National Institutes of Health USDoHaHS. Health Information National Trends Survey. 2013. http://hints.cancer.gov/docs/Instruments/HINTS_4_Cycle_3_English_Annotated_508c_3_21_2014.pdf (accessed July 2016).

22 Yaqub O, Castle-Clarke S, Sevdalis N, et al. Attitudes to vaccination: A critical review. *Soc Sci Med* 2014;112:1-11.

For peer review only - http://bmjopen.bmj.com/site/about/guidelines.xhtml

23 Bouder F, Way D, Löfstedt R, Evensen D. Transparency in Europe: A Quantitative Study. *Risk Anal* 2015;35:1210-29.

24 Eisma R, Dickinson A, Goodman J, et al. Early user involvement in the development of information technology-related products for older people. *Univers Access Inform Soc* 2004;3:131-40.

25 Public Health England. Vaccine coverage estimate for the GP based catch-up meningococcal ACWY (MenACWY) immunisation programme for school leavers (becoming 18 before 31 August 2016) in England, cumulative data to the end of March 2017. *Health Protection Report* 2017;11:1-4.

For peer review only

For peer review only - http://bmjopen.bmj.com/site/about/guidelines.xhtml

Supplementary File. Questionnaire. Items excluded (q 34-36; 39-41) as they were not designed for the current study.

Influences on students' intention to vaccinate

The NHS recommends a number of vaccinations for individuals of different ages and high-risk groups, free of charge. The NHS childhood vaccination programme offers a range of vaccines for children from 2 months up until they are 18. These vaccines protect children against vaccine-preventable diseases such as measles, human papillomavirus (HPV) and polio. People in the UK are also eligible for some free travel vaccines if they are visiting high-risk areas of the world.

We are interested in understanding students' attitudes towards vaccination.

Please read each of the statements below and show how much you agree or disagree with it by ticking the appropriate box.

* 2. Vaccinations are effective in preventing disease.

Strongly disagree	Disagree	Neither agree nor disagree	Agree	Strongly agree
○	○	○	○	○

* 3. I am concerned about vaccination side effects.

Strongly disagree	Disagree	Neither agree nor disagree	Agree	Strongly agree
○	○	○	○	○

* 4. Vaccinations are helpful.

Strongly disagree	Disagree	Neither agree nor disagree	Agree	Strongly agree
○	○	○	○	○

* 5. I am afraid of vaccinations.

Strongly disagree	Disagree	Neither agree nor disagree	Agree	Strongly agree
○	○	○	○	○

* 6. There are more reasons to have vaccinations than not to have vaccinations.

Strongly disagree	Disagree	Neither agree nor disagree	Agree	Strongly agree
○	○	○	○	○

* 7. I have a responsibility to receive vaccinations for the protection of others.

Strongly disagree	Disagree	Neither agree nor disagree	Agree	Strongly agree
○	○	○	○	○

* 8. People who don't get vaccinated put others at risk.

Strongly disagree	Disagree	Neither agree nor disagree	Agree	Strongly agree
○	○	○	○	○

* 9. More people should be vaccinated against diseases so that outbreaks do not occur.

Strongly disagree	Disagree	Neither agree nor disagree	Agree	Strongly agree
○	○	○	○	○

* 10. Vaccinating is doing the right thing.

Strongly disagree	Disagree	Neither agree nor disagree	Agree	Strongly agree
○	○	○	○	○

* 11. Vaccination is one way to make a positive contribution to your health.

Strongly disagree	Disagree	Neither agree nor disagree	Agree	Strongly agree
○	○	○	○	○

Influences on students' intention to vaccinate

Certain travel vaccinations are available free on the NHS. These vaccines immunize people against certain diseases prevalent in parts of the world (such as typhoid, hepatitis A and cholera). Imagine you are planning to travel to a part of Asia where typhoid is prevalent. Typhoid fever is a bacterial infection that can spread throughout the body affecting many organs. It can be fatal without prompt treatment. A typhoid vaccine is recommended and is available free on the NHS, usually from GP surgeries. You can find out more about typhoid fever and the vaccine here: http://www.nhs.uk/Conditions/Typhoid-fever/Pages/Introduction.aspx.

We will ask you some questions about the typhoid vaccine.

* 12. Have you had the typhoid vaccine in the last 3 years?*(a typhoid booster vaccination is recommended every 3 years if you continue to be at risk of infection).*

- ◯ Yes
- ◯ No
- ◯ Don't know

If *yes*, please imagine that you needed the vaccine for the following questions.

We are interested in what you think the views of others would be.

* 13. My immediate family members would think I should get the vaccine.*For example your Mum, Dad, brothers or sisters.*

Agree (1)	2	3	4	5	6	Disagree (7)
◯	◯	◯	◯	◯	◯	◯

* 14. My immediate family members would approve of the vaccine.*For example your Mum, Dad, brothers or sisters.*

Agree (1)	2	3	4	5	6	Disagree (7)
◯	◯	◯	◯	◯	◯	◯

* 15. Most of my family members would get the vaccine if it was recommended for them*For example your Mum, Dad, brothers or sisters.*

Likely (1)	2	3	4	5	6	Unlikely (7)
◯	◯	◯	◯	◯	◯	◯

* 16. My friends would think I should get the vaccine.

Agree (1)	2	3	4	5	6	Disagree (7)
◯	◯	◯	◯	◯	◯	◯

* 17. My friends would approve of the vaccine.

Agree (1)	2	3	4	5	6	Disagree (7)
○	○	○	○	○	○	○

* 18. Most of my friends would get the vaccine if it was recommended for them.

Likely(1)	2	3	4	5	6	Unlikely (7)
○	○	○	○	○	○	○

* 19. My GP would think I should get the vaccine.

Agree (1)	2	3	4	5	6	Disagree (7)
○	○	○	○	○	○	○

* 20. My GP would approve of the vaccine.

Agree (1)	2	3	4	5	6	Disagree (7)
○	○	○	○	○	○	○

* 21. My GP would get the vaccine if it was recommended for him/her.

Likely (1)	2	3	4	5	6	Unlikely (7)
○	○	○	○	○	○	○

Influences on students' intention to vaccinate

We're also interested in how *in control* you would feel about getting the typhoid vaccine.

* 22. Whether or not I got the vaccine would be entirely up to me.

Strongly disagree (1)	2	3	4	5	6	Strongly agree (7)
○	○	○	○	○	○	○

* 23. How much do you feel that whether you got the vaccine would be beyond your control?

Not at all (1)	2	3	4	5	6	Very much so (7)
○	○	○	○	○	○	○

Influences on students' intention to vaccinate

* 24. I believe I would have the ability to get the vaccine.

Definitely do not (1)	2	3	4	5	6	Definitely do (7)
○	○	○	○	○	○	○

* 25. To what extent do you see yourself as being capable of getting the vaccine?

Very unlikely (1)	2	3	4	5	6	Very likely (7)
○	○	○	○	○	○	○

* 26. How confident are you that you would be able to get the vaccine?

Very unsure (1)	2	3	4	5	6	Very sure (7)
○	○	○	○	○	○	○

* 27. If it were entirely up to me, I am confident that I would be able to get the vaccine.

Strongly disagree (1)	2	3	4	5	6	Strongly agree (7)
○	○	○	○	○	○	○

Influences on students' intention to vaccinate

Please tick the box that applies most to you.

* 28. Did you have all the recommended vaccines as a child?

- I had none of the recommended vaccines as a child
- I had a few of the recommended vaccines as a child
- I had most of the recommended vaccines as a child
- I had all of the recommended vaccines as a child
- I don't know what vaccines I had as a child

* 29. Have you ever had a bad reaction to a vaccination?

- Yes
- No
- Don't know

Influences on students' intention to vaccinate

* 30. If *yes*, how serious was your reaction?

- Required medical treatment
- Uncomfortable for more than 1 day (no medical treatment)
- Uncomfortable for less than one day (no medical treatment)
- I don't know

Influences on students' intention to vaccinate

* 31. Are you scared of needles?

Not at all (1)	2	3	4	Very scared (5)
○	○	○	○	○

Influences on students' intention to vaccinate

* 32. If I were recommended a vaccine, I would try to get it.

Strongly disagree (1)	2	3	4	Strongly agree (5)
○	○	○	○	○

* 33. If I were recommended a vaccine, I would intend to get it.

Strongly disagree (1)	2	3	4	Strongly agree (5)
○	○	○	○	○

Influences on students' intention to vaccinate

Now we'd like to ask you some more general questions about your health.

Imagine you had a problem with your health.

37. Would you use any of these sources for information? For sources that you would use, please specify the name of the source. For example, for the internet, you might use the NHS website.

Books	
Brochures, pamphlets	
Family	
Friend/Co-worker	
Doctor or health care provider	
Internet	
Library	
Magazines	
Newspapers	
Telephone information number	
Complementary, alternative, or unconventional practitioner	
Other	

Imagine you have been recommended to have a vaccination as part of the UK Immunisation Schedule.

38. Would you use any of these sources for information on the vaccine? For sources that you would use, please specify the name of the source. For example, for the internet, you might use the NHS website.

Books	
Brochures, pamphlets	
Family	
Friend/Co-worker	
Doctor or health care provider	
Internet	
Library	
Magazines	
Newspapers	
Telephone information number	
Complementary, alternative, or unconventional practitioner	
Other	

Influences on students' intention to vaccinate

We are now going to ask some demographic questions.

* 42. What is your age?

* 43. What is your gender?

- Male
- Female
- Prefer not to say

* 44. Do you have any children?

- Yes
- No
- Prefer not to say

* 45. What is your ethnic group? (Please tick one box)

- White British
- White other *(please specify below)*
- Asian British
- Asian other *(please specify below)*
- Black British
- Black other *(please specify below)*
- Mixed ethnicity British *(please specify below)*
- Mixed ethnicity other *(please specify below)*
- Arab
- Other ethnic group *(please specify below)*
- Prefer not to say

Please specify here, if asked

* 46. Where did you live until the age of 4?

- UK
- Not UK *(please specify below)*
- Both *(please specify below)*

Please specify here, if asked

* 47. Do you have a religion?

- No religion
- Christian (including Church of England, Catholic, Protestant and all other Christian denominations)
- Buddhist
- Hindu
- Jewish
- Muslim
- Sikh
- Prefer not to say
- Any other religion (please specify)

Think of this ladder as representing where people stand in the UK.

At the *top* of the ladder are the people who are best off – those who have the most money, the most education and the most respected jobs. At the *bottom* are the people who are the worst off – who have the least money, least education, and the least respected jobs or no job. The higher up you are on this ladder, the closer you are to the people at the very top; the lower you are, the closer you are to the people at the very bottom.

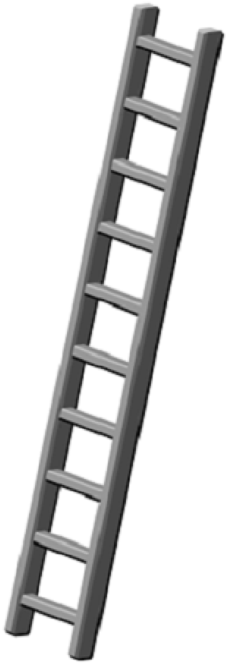

* 48. Now think about your family. Please tell us where you think your family would be on this ladder.

Please tick the box next to the rung that best represents where your family would be on this ladder.

- Top: Rung 10
- Rung 9
- Rung 8
- Rung 7
- Rung 6
- Rung 5
- Rung 4
- Rung 3
- Rung 2
- Bottom: Rung 1

* 49. Where would you place yourself on this ladder?

Please tick the box next to the rung where you think you stand at this time in your life, relative to other people in the UK.

- Top: Rung 10
- Rung 9
- Rung 8
- Rung 7
- Rung 6
- Rung 5
- Rung 4
- Rung 3
- Rung 2
- Bottom: Rung 1

STROBE Statement—checklist of items that should be included in reports of observational studies

	Item No	Recommendation	Page No
**Title and abstract**	1	(*a*) Indicate the study's design with a commonly used term in the title or the abstract	1
		(*b*) Provide in the abstract an informative and balanced summary of what was done and what was found	3
**Introduction**			
Background/rationale	2	Explain the scientific background and rationale for the investigation being reported	4
Objectives	3	State specific objectives, including any prespecified hypotheses	4
**Methods**			
Study design	4	Present key elements of study design early in the paper	5
Setting	5	Describe the setting, locations, and relevant dates, including periods of recruitment, exposure, follow-up, and data collection	5
Participants	6	(*a*) *Cohort study*—Give the eligibility criteria, and the sources and methods of selection of participants. Describe methods of follow-up<br>*Case-control study*—Give the eligibility criteria, and the sources and methods of case ascertainment and control selection. Give the rationale for the choice of cases and controls<br>*Cross-sectional study*—Give the eligibility criteria, and the sources and methods of selection of participants	5
		(*b*) *Cohort study*—For matched studies, give matching criteria and number of exposed and unexposed<br>*Case-control study*—For matched studies, give matching criteria and the number of controls per case	N/A
Variables	7	Clearly define all outcomes, exposures, predictors, potential confounders, and effect modifiers. Give diagnostic criteria, if applicable	5
Data sources/ measurement	8*	For each variable of interest, give sources of data and details of methods of assessment (measurement). Describe comparability of assessment methods if there is more than one group	5
Bias	9	Describe any efforts to address potential sources of bias	Not included
Study size	10	Explain how the study size was arrived at	5
Quantitative variables	11	Explain how quantitative variables were handled in the analyses. If applicable, describe which groupings were chosen and why	5
Statistical methods	12	(*a*) Describe all statistical methods, including those used to control for confounding	6
		(*b*) Describe any methods used to examine subgroups and interactions	6
		(*c*) Explain how missing data were addressed	Not included
		(*d*) *Cohort study*—If applicable, explain how loss to follow-up was addressed<br>*Case-control study*—If applicable, explain how matching of cases and controls was addressed<br>*Cross-sectional study*—If applicable, describe analytical methods taking account of sampling strategy	N/A
		(*e*) Describe any sensitivity analyses	6

Continued on next page

For peer review only - http://bmjopen.bmj.com/site/about/guidelines.xhtml

**Results**			
Participants	13*	(a) Report numbers of individuals at each stage of study—eg numbers potentially eligible, examined for eligibility, confirmed eligible, included in the study, completing follow-up, and analysed	5
		(b) Give reasons for non-participation at each stage	N/A
		(c) Consider use of a flow diagram	N/A
Descriptive data	14*	(a) Give characteristics of study participants (eg demographic, clinical, social) and information on exposures and potential confounders	6
		(b) Indicate number of participants with missing data for each variable of interest	N/A
		(c) *Cohort study*—Summarise follow-up time (eg, average and total amount)	N/A
Outcome data	15*	*Cohort study*—Report numbers of outcome events or summary measures over time	N/A
		Case-control study—Report numbers in each exposure category, or summary measures of exposure	N/A
		Cross-sectional study—Report numbers of outcome events or summary measures	6/7
Main results	16	(*a*) Give unadjusted estimates and, if applicable, confounder-adjusted estimates and their precision (eg, 95% confidence interval). Make clear which confounders were adjusted for and why they were included	6-8
		(*b*) Report category boundaries when continuous variables were categorized	5
		(*c*) If relevant, consider translating estimates of relative risk into absolute risk for a meaningful time period	N/A
Other analyses	17	Report other analyses done—eg analyses of subgroups and interactions, and sensitivity analyses	6
**Discussion**			
Key results	18	Summarise key results with reference to study objectives	10/11
Limitations	19	Discuss limitations of the study, taking into account sources of potential bias or imprecision. Discuss both direction and magnitude of any potential bias	11
Interpretation	20	Give a cautious overall interpretation of results considering objectives, limitations, multiplicity of analyses, results from similar studies, and other relevant evidence	11
Generalisability	21	Discuss the generalisability (external validity) of the study results	11
**Other information**			
Funding	22	Give the source of funding and the role of the funders for the present study and, if applicable, for the original study on which the present article is based	12

*Give information separately for cases and controls in case-control studies and, if applicable, for exposed and unexposed groups in cohort and cross-sectional studies.

Note: An Explanation and Elaboration article discusses each checklist item and gives methodological background and published examples of transparent reporting. The STROBE checklist is best used in conjunction with this article (freely available on the Web sites of PLoS Medicine at http://www.plosmedicine.org/, Annals of Internal Medicine at http://www.annals.org/, and Epidemiology at http://www.epidem.com/). Information on the STROBE Initiative is available at www.strobe-statement.org.

For peer review only
